# Epidemiology of Astrovirus, Norovirus and Sapovirus in Greek pig farms indicates high prevalence of Mamastrovirus suggesting the potential need for systematic surveillance

**DOI:** 10.1186/s40813-021-00245-8

**Published:** 2022-01-09

**Authors:** Efthymia Stamelou, Ioannis A. Giantsis, Konstantinos V. Papageorgiou, Evanthia Petridou, Irit Davidson, Zoe S. Polizopοulou, Anna Papa, Spyridon K. Kritas

**Affiliations:** 1 School of Veterinary Medicine, Faculty of Health Sciences, Aristotle University of Thessaloniki, 54124 Thessaloniki, Greece; 2 Department of Animal Science, Faculty of Agricultural Sciences, University of Western Macedonia, 53100 Florina, Greece; 3 Kimron Veterinary Institute, 50250 Bet Dagan, Israel; 4 Laboratory of Microbiology, School of Medicine, Faculty of Health Sciences, Aristotle University of Thessaloniki, 54124 Thessaloniki, Greece

**Keywords:** Astrovirus, Norovirus, Sapovirus, Kobuvirus, Sapelovirus, Pigs, Swine, Greece

## Abstract

**Backround:**

Astrovirus, Norovirus and Sapovirus exhibit a wide distribution in swine pig herds worldwide. However, the association of porcine Astrovirus (PAstV), porcine Norovirus (PoNoV) and porcine Sapovirus (PoSaV) with disease in pigs remains uncertain. In this study, we investigated the prevalence of PAstV, PoNoV and PoSaV in Greek pig farms using both conventional RT-PCR and SYBR-Green Real-time RT-PCR in an effort to compare the sensitivity of the two methods. We examined 1400 stool samples of asymptomatic pigs originating from 28 swine farms throughout Greece in pools of five.

**Results:**

PAstV was detected in all 28 swine farms examined, with an overall prevalence of 267/280 positive pools (95.4%). Porcine Caliciviruses prevalence was found at 36 and 57 out of the 280 examined samples, by the conventional and SYBR-Green Real time RT-PCR, respectively. Sequencing and phylogenetic analysis of the positive samples revealed that the detected PAstV sequences are clustered within PAstV1, 3 and 4 lineages, with PAstV3 being the predominant haplotype (91.2%). Interestingly, sequencing of the Calicivirus positive samples demonstrated the presence of non-target viruses, i.e. Sapovirus, Kobuvirus and Sapelovirus sequences and one sequence highly similar to bat Astrovirus, while no Norovirus sequence was detected.

**Conclusions:**

The high prevalence of PAstV in Greek pig farms poses a necessity for further investigation of the pathogenicity of this virus and its inclusion in surveillance programs in case that it proves to be important. To our knowledge, this is the first epidemiological study of these viruses in pig farms in Greece.

**Supplementary Information:**

The online version contains supplementary material available at 10.1186/s40813-021-00245-8.

## Background

Astrovirus, Norovirus and Sapovirus are small, round-structured, single-stranded, positive sense RNA viruses. They are considered as enteric pathogens that can cause diarrhea in a wide variety of animals, such as humans, pigs, dogs, cats, mink and a lot of avian species [1–5]). They have a worldwide distribution, whereas Noroviruses (NoVs) and Sapoviruses (SaVs) have been characterized as the most common cause of viral gastroenteritis in humans worldwide [2].

Norovirus and Sapovirus are members of the family Caliciviridae. They are both non-enveloped, single-stranded, positive-sense RNA viruses of 7.3 to 8.5 kb in size [6]. Based on the genome structure, the Caliciviridae can be further differentiated into two groups [7]. In the first, including the Norovirus, the open reading frame 1(*ORF1)* is separated from *ORF2* and *ORF3* near the 3’ end, whereas an *ORF4* (comprised within *ORF2*) encodes the virulence factor, *VF1*. In the second, containing the Sapovirus, there is a large *ORF1* and a standard *ORF2* (equivalent to the *ORF3* of the Norovirus), whereas an *ORF3* has been suggested as equivalent to *ORF4* [7] Norovirus and Sapovirus are classified into genogroups and genotypes based on the major structural capsid protein (*VP1*) sequence. Norovirus is organized into at least seven genogroups (GI-GVII), three of which, i.e. GI, GII and GIV viruses, have been detected in humans [8]. Sapovirus is classified in 19 genogroups (GI-GVIII), four of which (GI, GII, GIV and GV) have been detected in humans [9, 10]. Porcine Sapoviruses (PoSaVs) belong to the genogroup GIII (strain A, B and C) and GVII [10]. Porcine Noroviruses belong to the genogroup GII and more specifically to three distinct GII P-types i.e. GII.P11, GII.P18 and GII.P19 [11–27]).

In swine, porcine Sapovirus has proved to cause intestinal disease by experimental infection [28, 29]. Sapoviruses have been detected in swine farms in numerous pig herd studies worldwide, both in asymptomatic and symptomatic pigs with diarrhea [20, 30–37]). More specifically, the highest prevalence was seen among piglets aged between 2 and 8 weeks, and there was no significant difference in the proportion of sapovirus-positive findings for healthy animals and animals with diarrhea. In Axel Mauroy’s et al. [20], the presence of Norovirus and Sapovirus in pigs in Belgium was investigated by examining 43 swine faecal samples from a veterinary diagnostic laboratory. PoSaVs were detected in 5/43 stool samples of both diarrhoeic and asymptomatic piglets, while Porcine NoVs were only detected in 2 pigs without clinical signs. PoNoV strains were detected in younger pigs (16–20 weeks). In Ilaria Di Bartolo et al. [15] in Italy, 201 fecal specimens from asymptomatic and 89 speciments from pigs with diarrhea were examined for the presence of porcine Caliciviruses and PoSaV was detected in 6.9% of the asymptomatic pigs and in, 18/89(20%) of the symptomatic, while PoNoV was detected in 1 asymptomatic pig. The highest prevalence of PoSaV was detected in the United States, were in a study of 621 fecal samples (11 with diarrhoea and the rest clinically normal) from pigs of various ages PoSaV was detected in 62% of the pigs with the highest rate being observed in nursery pigs and lowest in sucklingpigs [26]. In the same study, PoNoV was detected in 20% of the finisher pigs. The lowest PoSaV prevalence has been observed in Taiwan, where the virus was detected in 0.57% (5/863) of asymptomatic pigs [16]. Thus, the role of Sapovirus in enteric disease in swine remains unclear. The worldwide presence of Sapovirus has been proved in many prevalence studies, but the detection rates vary a lot, ranging from 3 to 67% [19, 26, 32, 35, 37, 38]. Generally, the highest prevalence of Sapovirus is detected in post-weaning pigs [30].

Porcine Noroviruses (PoNoVs) have been detected mainly in asymptomatic adult pigs [25, 39]). GII PoNoVs have been detected in pigs in the USA, in Latin America, and in several European countries, in both symptomatic and asymptomatic animals [11, 17, 18, 20, 21, 40]. GI and GII NoVs have been detected in swine fecal samples, as well as in retail and imported raw meat samples [16, 41, 42]. This fact has raised public health concerns regarding the zoonotic potential of porcine NoVs and the role of swine in the epidemiology of this infection, owing to the possibility of emergence of new viral recombinant strains that can be transmitted directly to humans [40, 41, 43, 44]. So far, the association of swine NoVs with human infections remains unclear and further research is needed in order for this virus infection to be elucidated or controlled [39].

Astroviruses belong to the family Astroviridae. They are small, approximately 28–30 nm in diameter, non-enveloped, and contain a + ssRNA genome approximately 6.4–7.7 kb in length [44]. The family Astroviridae is divided into two genera, Mamastrovirus (19 species) and Avastrovirus (3 species) [45]. Members of the genus Mamastrovirus infect various mammals, including human [46], bovine [47], feline [48], porcine [49] and mink [50]. Members of the genus Avastrovirus mainly infect avian species such as chicken, turkey, and duck [51–53].

Porcine astroviruses (PAstVs) belong to the Mamastrovirus genus. The first identification of PAstV took place in 1980, from fecal samples of diarrheal pigs, by the means of electron microscopy [54]. To date, five genotypes of PAstVs have been identified [55]. The genome of PAstV encodes for three open reading frames (*ORFs*), namely *ORF1a, ORF1b,* and *ORF2* [56]. ORF1a and ORF1b encode the non-structural proteins and an RNA-dependent RNA polymerase *(RdRp*), while *ORF2* encodes for the viral capsid structural proteins [45]. PAstVs are responsible for gastrointestinal disease, mainly in young individuals and have been detected in the intestines and faeces of pigs. Regarding the age group detection, the highest detection rate of PAstV across different studies has been noted in boars (82%), followed by nursery pigs (67%), 59% in finisher pigs, 36% in gilts, 37% in sows, and 22% in suckling piglets [57]. However, some porcine astroviruses have been detected in pigs with extra-intestinal manifestations, such as respiratory and neurological signs [58]. Additionally, PoAstVs have been detected in asymptomatic pigs [59]. Thus, the role of PAstV in disease remains unclear.

**Fig. 1:**
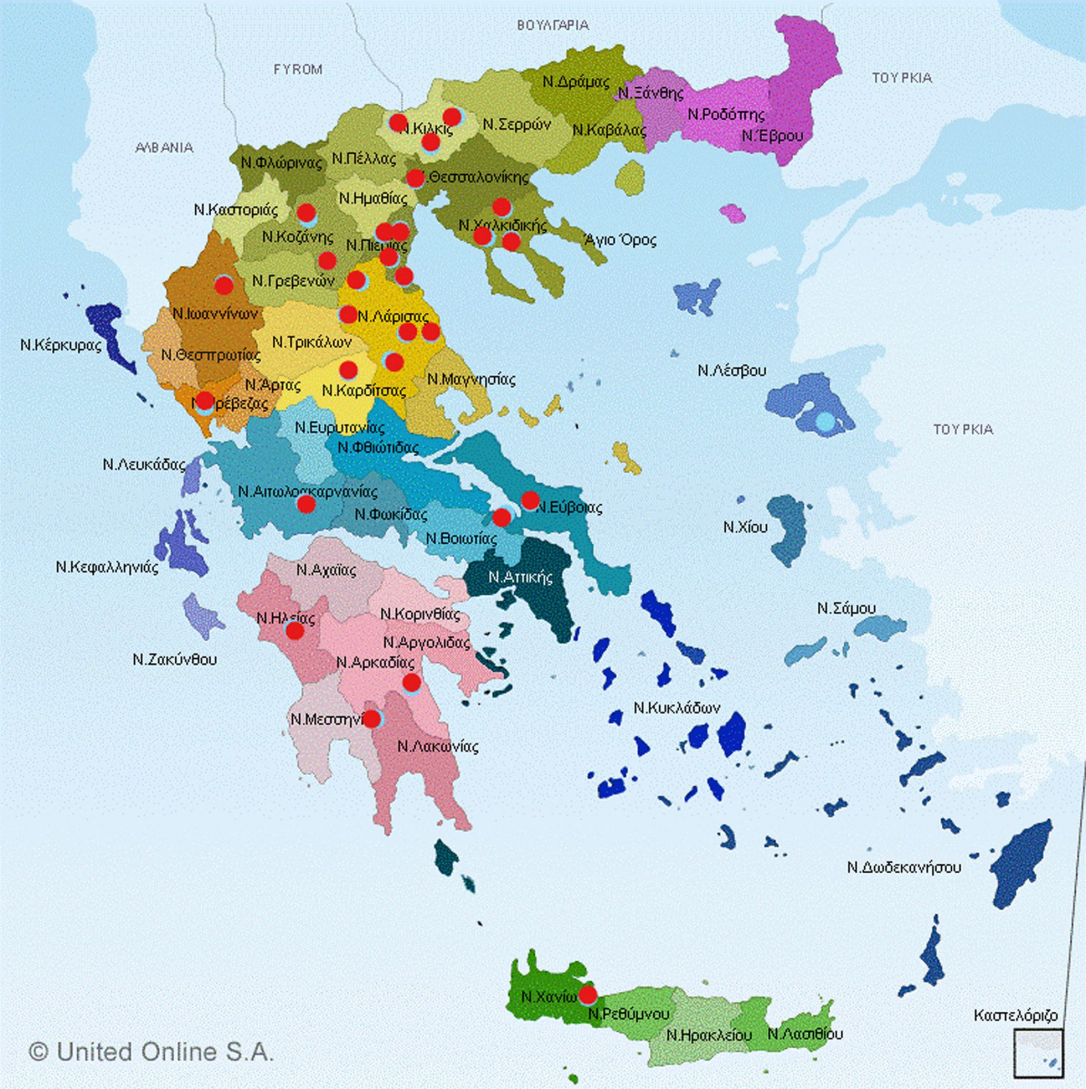
Map of Greece containing the areas of sample collection, indicated with red dots

The main goal of the present study was to examine the epidemiology of Astrovirus, Norovirus and Sapovirus in pig farms throughout Greece (Fig. 1). For this purpose, the faeces of 1400 pigs originating from 28 Greek swine farms were investigated for the presence of these three viruses applying molecular assays. Also, a secondary scope was to investigate the different five age groups, i.e. suckling, nursery, grower, finishing pigs and sows. All samples originated from asymptomatic animals. The samples were divided in totally 280 pools of five samples from the same pig farm each pool. In an effort to evaluate two different molecular techniques in terms of sensitivity, two different RT-PCR methods were used: a conventional and a SYBR-Green real-time RT-PCR, in order to compare their sensitivity and specificity in the detection of these 3 RNA viruses. Finally, scoping to phylogenetically analyze the positive samples, sequencing was performed that was followed by the construction of phylogenetic trees.

## Results

### Astrovirus

**Fig. 2:**
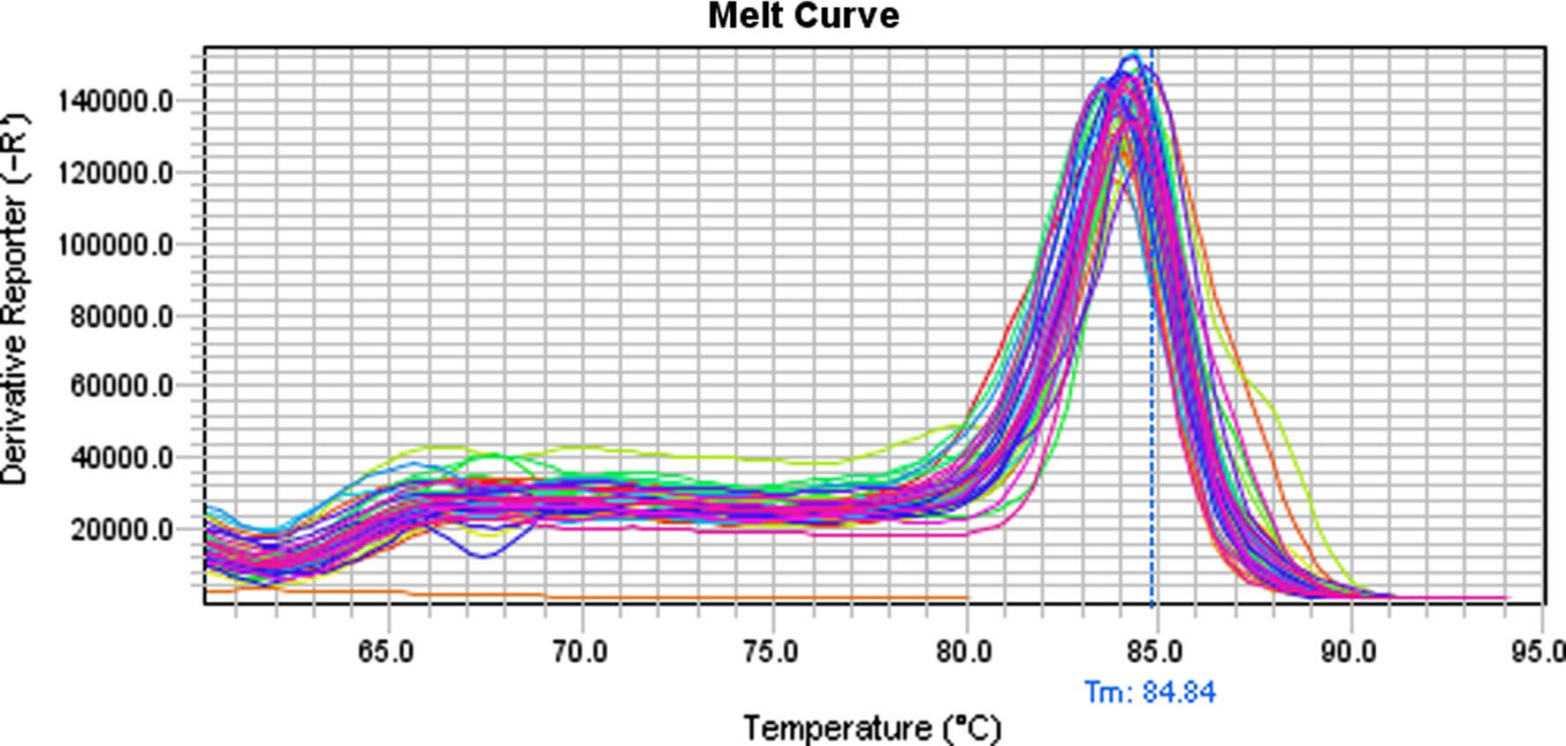
Melting curve profiles of the examined porcine samples. Each peak indicates one different analysed amplicon

Both methodologies applied, RT-PCR and real time RT-PCR, detected 267 positive pools out of the 280 (95.4%) samples examined (Fig. 2). Thus, concerning sensitivity, the two different methods were of equal sensitivity. At farm level, all farms investigated were positive to Astrovirus (28/28, 100%). The distribution of the virus on the different porcine age groups was the following: 100% (56/56 pools) of the nursery and grower pigs, 96.4% (54/56 pools) of the suckling piglets, 94.6% (53/56) of the finishing pigs, and 98.2% (55/56) of the sows were positive to the virus.

#### Norovirus and Sapovirus

Caliciviruses were detected in 36/280 (12.9%) positive pools of the pigs using universal calici-primers p289-p290 with the method of conventional RT-PCR and in 57/280 (20.4%) of the pigs with the method of SYBR-Green real time RT-PCR, indicating a greater sensitivity SYBR-Green real time RT-PCR. The age group distribution of these viruses based on conventional RT-PCR results is the following: 6/56 (10.7%) at suckling pigs, 5/56 (8.9%) at nursery pigs, 7/56 (12.5%) at grower pigs, 17/56 (30.4%) at finishing pigs and 1/56 (1.8%) at sows. On the other hand, based on the results of the SYBR-Green real time RT-PCR, Caliciviruses were detected in 9/56 (16.1%) of suckling pigs, 5/56 (8.9%) of nursery pigs, 6/56 (10.7%) of grower pigs, 36/56 (64.3%) of finishing pigs and 1/56 (1.8%) of the sows.

### Sequencing results

#### Astrovirus

**Fig. 3:**
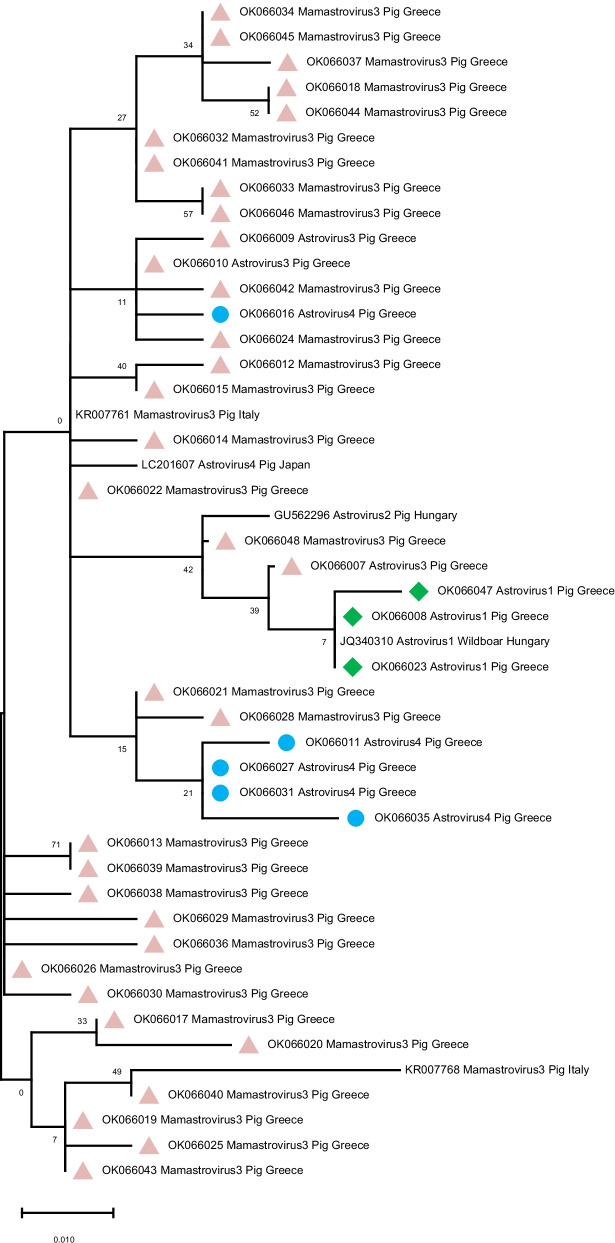
Phylogenetic analysis of nucleotide sequences from the *ORF1b* gene (183-bp fragment) of 47 PAstV strains detected in this study and 5 PAstV reference sequences. The sequences that correspond to PAstV3, detected in the present study, are indicated with pink triangles (  ). The sequences of PAstV1 detected in the present study are indicated with green rhomb (  ) and the sequences of PAstV4 are indicated with blue circles (  ). GenBank accession numbers are shown on the tree

**Fig. 4:**
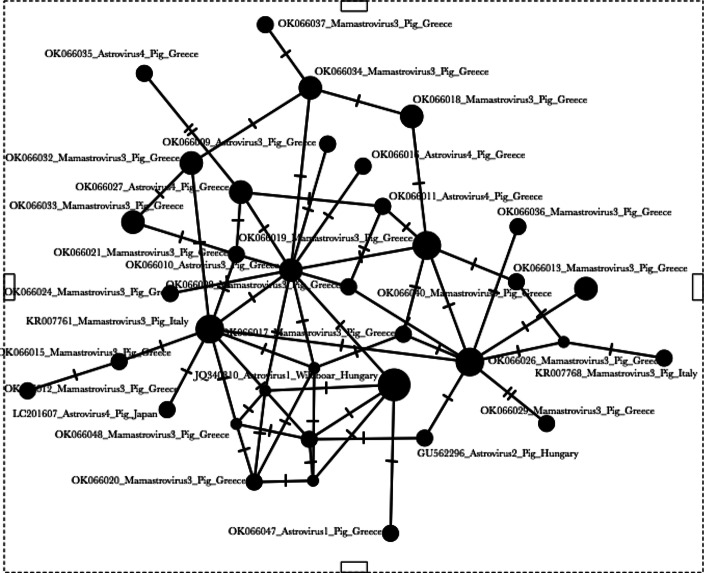
Median-joining haplotype network of the Astrovirus sequences. Circle sizes are proportional to the frequency of each haplotype, whereas the unlabeled dots indicate interval missing haplotypes, potentially not sampled, known as median vectors

Ninety-five out of the 267 positive samples were sent for sequencing comprising a representative subsample towards validation of the results, as well as in the direction of phylogenetic analysis. Of these, 91 sequences had over 90% similarity with Astrovirus strains based on the blast tool. In 83/91 (91.2%) of the sequenced samples a sequence similarity over 90% with Mamastrovirus type 3 strain was revealed. In 2/91 (2.2%) of the sequenced samples, sequence similarity greater than 97% with Astrovirus type 4 strains was estimated. In two samples, sequences of approximately 95% sequence similarity with Astrovirus type 1 were determined based on the blast tool usage for genetic similarity assessment. The 95 sequences defined 42 haplotypes that were deposited in the GenBank database (Accession Numbers: OK066007–OK066048, Additional file 1), the phylogenetic relationships of which, in comparison with haplotypes retrieved from GenBank are shown in Fig. 3. Additionally, evolutionary relationships of these haplotypes are shown in the median-joining network of Fig. 4. The network was occupied by a central haplogroup, arranged in a star-like manner, with multiple reticular linkages connecting the central haplotypes, probably constituting the common ancestors of the Astrovirus strains.

#### Norovirus and Sapovirus

**Fig. 5:**
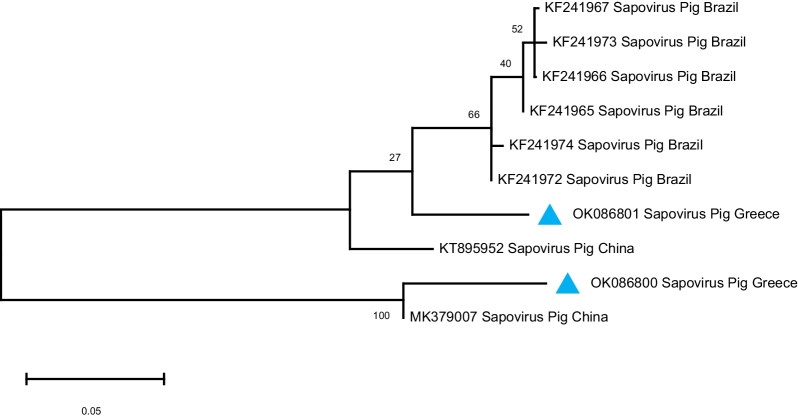
Phylogenetic tree based on partial RNA dependent RNA polymerase coding region (331 bp) of porcine Sapovirus strains identified in this study and porcine Sapovirus reference strains. The tree was created with maximum likelihood method of MEGA program. The strains that were detected in our study are indicated with blue trianges (  ). GenBank accession numbers are shown on the tree

**Fig. 6:**
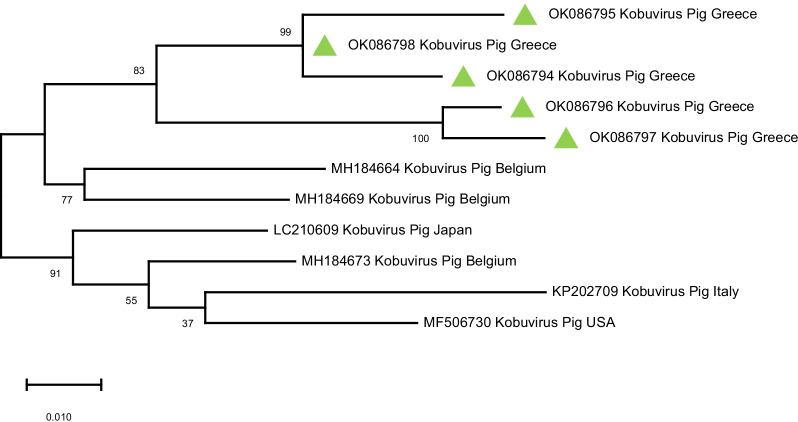
Phylogenetic analysis of porcine Kobuvirus sequences detected with primers p289-p290, targeting the *RdRp* region of porcine Caliciviruses. The sequences detected in the present study are indicated with green triangles (  ). Porcine Kobuvirus reference sequences obtained from GenBank were included in the phylogenetic tree. GenBank accession numbers are shown on the tree

**Fig. 7:**
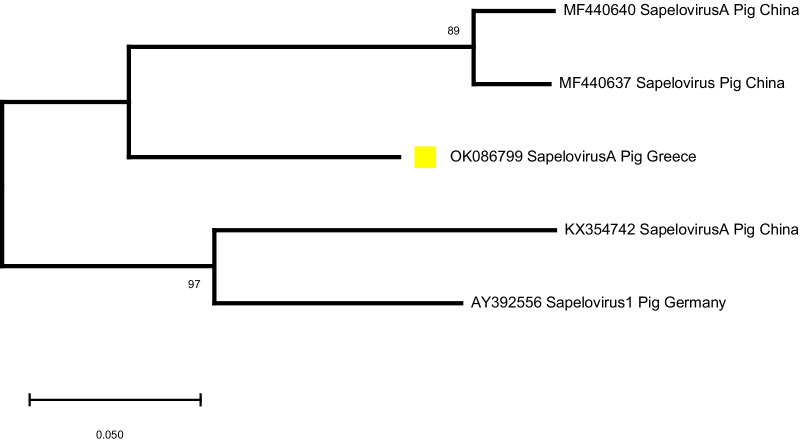
Phylogenetic analysis of porcine Sapelovirus sequences detected with primers p289–p290, targeting the *RdRp* region of porcine Caliciviruses. The sequences detected in the present study are indicated with yellow squares (  ). Porcine Sapelovirus reference sequences obtained from GenBank were included in the phylogenetic tree. GenBank accession numbers are shown on the tree

Of the 36 Calicivirus-positive samples (with the method of conventional RT-PCR), 10 samples resulted in sequences that had a similarity with certain viruses (Sapovirus, Kobuvirus and Sapelovirus) when assessed with the blast tool, and eventually defined 8 haplotypes (see Fig. 5). These sequences were deposited in the GenBank database and assigned the accession numbers OK086794-OK086801. Two sequences shared approximately 95% similarity with porcine Sapovirus GVII and GIII. In 5 samples there were detected sequences that based on blast searches had over 95% similarity with Kobuvirus. Among the three remaining samples, 2 were very closely related with Sapelovirus sequences, whereas in one sample a sequence similarity of 92% with bat Astrovirus was determined. Phylogenetic relationships of those haplotypes with corresponding ones obtained from the GenBank database are shown in Figs. 5, 6 and 7. No sequence of porcine Norovirus was detected.

More specifically, the two Sapovirus sequences detected by sequencing were compared using the BLAST tool in order to search for similar sequences from other studies. One Sapovirus sequence (X23AP10, GB acc. number OK086800) showed high similarity with Porcine sapovirus isolate PoSaV_VIRES_NM01_C3 polyprotein gene (MK379007, Fig. 5). The second Sapovirus sequence detected in our study (X13PRO2, GB acc.number OK086801) showed 93% similarity with Porcine enteric Sapovirus strain swine/GVII.1/SUI-101/2008/PA/BRA RNA dependent RNA polymerase gene (KF241967).

## Discussion

### Astrovirus

In the present study we investigated the presence of Astrovirus in 1400 pigs of 5 different age groups originating from 28 different pig farms around Greece. The primer pair PAstVDF-R was selected for the detection of porcine Astrovirus due to its ability to simultaneously detect all of the five porcine Astrovirus types. As noted previously, these primers target a conserved region within *ORF1b* of Astrovirus genome, allowing in this way the detection of all PAstV types. In Xiao ‘s et al. [60] research, five different TaqMan probes (one for the detection of each of the five Astrovirus types) were used along with the primers in two different multiplex qRT-PCR assays, one with probes for Astrovirus type 1 and 2 and one with the probes for Astrovirus type 3, 4 and 5. In our research, a different approach was followed. The PAstVDF-PAstVDR primers were used in conventional and SYBR-Green real time RT-PCR for the detection of porcine Astrovirus and the positive samples were sequenced in order to validate the results, determine the Astrovirus type, strain and phylogenetically characterize the position of the strains. Both RT-PCR methods used for the detection of PAstV showed similar sensitivity, with the SYBR GREEN RT-PCR being slightly more sensitive. The melting temperature of PAstV- positive samples was approximately 85 °C (Fig. 2).

More specifically, based on the conventional and SYBR-Green real time RT-PCR results, we see that Astrovirus is widely distributed in Greek swine farms (100% of the farms were positive to the virus). Porcine Astrovirus was detected in all five age groups examined with similar proportions i.e. 100% of the nursery and grower pigs, 96.4% of the suckling, 94.6% of the finishing pigs and 98.2% of the sows. PAstV prevalence varies a lot from county to country and at different studies within the same country, with a detection rate ranging from 2.82% in China [61] to 94.4% (in healthy pigs) in Slovakia [62]. With respect to the different age groups, the frequency of detection of PoAstVs ranges from 0 to 100% in pigs of all ages, from suckling to adults [37, 55, 58, 61, 63–72].

Furthermore, based on the sequencing results, Mamastrovirus type 3 is the predominant Astrovirus type in the Greek swine farms (91.2%). However, sequences of Astrovirus type 1, and 4 were also detected (Fig. 3). Based on the blast tool for genetic similarity assessment as well as the phylogenetic analysis, we see that the sequences obtained from the samples of our study show close genetic similarity with PAstV sequences from Italy (PAstV3), Hungary (PAstV2, Wild Boar Astrovirus 1) and Japan (PAstV4) (Fig. 3). This is peculiar considering that breeding animals in Greece are rarely imported from Hungary or Italy and definitely never outside European Union e.g. Japan. Therefore, direct transmission by pig transport from such countries can be surely excluded. Neither wild boars from these countries are expected to move for such long distances. It cannot be excluded though that pigs imported from other main supplier countries (e.g. France, Germany, Denmark, Holland) did not carry this microorganism from the country of origin. Nor it can be excluded that imported feed ingredients included in large quantities in animal feeds (e.g. soybean) could be infected with such viruses at their collection point. Soybean, for instance, is mainly imported in EU from USA, although substantial quantities are imported from Brazil and China as well. Besides, viruses with many hosts are expected to exhibit low levels of genetic distance and thus may not represent really different strains. As these microbes were not associated so far with the occurrence of any disease, no thorough investigation for such microorganisms has been performed. The genetic homogeneity and absence of genetic isolation by distance, nor geographic structuring, is also in line with previous analyses of the same viruses, as indicated by the retrieved sequences from the Genbank database that originate from different countries indicating a genetic admixture pattern. This pattern of dispersal over large distances and no barriers to gene flow, suggests that the majority of the Astrovirus strains share a common origin, as demonstrated by the haplotype network of Fig. 4 as well, with several central haplotypes linked to each other.

In Xiao et al.’s [60] study, PAstV4 was the main Astrovirus type detected (62.3%), while PAstV3 was detected in solely 1.2% of the samples (the lowest percentage of all the other PAstV types). At Yifeng Qin et al.’s study (2019) [73], PAstV2 was the dominant Astrovirus type (44.4%), while PAstV3 was detected in the smallest percentage as well (only 1.4%). Moreover, at Zhou et al.’s study (2016) [74] including pigs from five European countries, PAstV4 was the only PAstV type detected. Nevertheless, our findings are in accordance with Rawal et al. [75], where three cross-sectional studies were carried out on sow farms located in the United States, with and without PAstV3-associated neurologic disease in the downstream nursery. In this study, PAstV3 was detected in very high rates varying from 66 to 90%, depending on the different age groups (highest frequency in sows and piglets). PAstV3 has been occasionally molecularly detected in swine nervous tissue [64, 68, 70, 71, 76–78] however, the neuropathogenic role of the virus remains to be clarified. Interestingly, in some of our samples mixed detection of Sapovirus, Kobuvirus or Sapelovirus was observed. It should be also emphasized that since all samples were collected from asymptomatic animals, they did not cause any decrease in productivity and the main risk associated with their detection is the implication as intermediate host for other animals to which the viruses are more harmful and cause illness. Particularly, the clinical significance of the detection of these viruses remains uncertain, as no specific clinical signs were noticed in the pig populations that we examined. Further investigation of the pathogenicity of this virus e.g. by challenge laboratory trials to investigate any deleterious effect on the pigs may be necessary in order to determine the importance of this virus. Thereafter, due to the predominant presence of PAstV in the Greek pig farms, systematic surveillance is recommended for this pathogen. It should be considered that PAstV is an RNA virus and therefore has a great ability to mutate, as well as new, more pathogenic strains could emerge, with zoonotic possibility.

## Norovirus and Sapovirus

The general Calicivirus primers p289–p290 allow the detection of a broad range of infections caused by Caliciviruses. This primer pair has been used for the detection of Caliciviruses in a wide variety of animals, such as swine, dogs, cats, chicken, turkeys, as well as humans [79–83]. However, p289–p290 lack specificity. This means that all positive RT-PCR samples with the conventional RT-PCR need sequencing in order to confirm the virus detected. In the present study, approximately 13% and 20% of the examined pools were positive to Caliciviruses with the conventional and SYBR Green RT-PCR, respectively. These results indicate an increased sensitivity of the SYBR-Green RT-PCR method, which is in agreement with the results of Mauroy’s et al. (2012) study [79]. The worldwide presence of Sapovirus has been proved in a lot of prevalence studies, but the detection rates vary a lot, ranging from 0.57 to 62% [16, 19, 26, 32, 35, 37, 38]. Our findings indicate a detection rate within those threshold values and particularly place the Sapovirus prevalence in Greek pig farms approximately on the average of the previous reports. Generally, the highest prevalence of Sapovirus is detected in nursery pigs [32]. More specifically, in Reuter et al.’s study (2010) [32], 1.050 swine fecal samples were examined for the presence of PoSaV, deriving from 88 pig farms in six European countries. PoSaV was detected in 7.6% of the samples. On the other hand, the lowest PoSaV detection rates were observed in China, where the virus was detected in 3.42% of 146 diarrheic stool samples of one-month-old piglets [84].

No sequence of Norovirus was detected in our samples. Interestingly, sequencing results in our study showed that primer pair p289–p290 also amplified viruses other than those of the Caliciviridae family. Particularly, apart from Sapovirus, a wide range of viruses was detected, including Astrovirus, Kobuvirus and Sapelovirus, the latter two of which were non-target microorganisms in our study. Kobuviruses (KoVs) are members of the family Picornaviridae, the order Picornavirales, and the genus Kobuvirus, one of 8 genera of the family. They are small, non-enveloped, round, single-stranded positive-sense RNA viruses with one large open reading frame encoding for a single polyprotein [85]. Within the genus Kobuvirus there are three distinct clusters. Aichivirus A (AiV-A) includes human AiV-1, canine KoV-1, and murine KoV-1. Aichivirus B (AiV-B) includes bovine KoV-1 and sheep KoV-1. Aichivirus C (AiV-C) includes porcine KoV-1 (PKoV/AiV-C) [85]. Porcine kobuvirus (PKoV) is a suspected cause of diarrhea in young piglets. PKoV has been found in feces from ill pigs, however, co-infection with other enteric viruses is common and may play a role in the clinical signs observed [86]. KoVs present a health hazard for humans. They have been isolated from shellfish, clams, oysters, and groundwater and are identified as a cause of foodborne illness [87]. No zoonotic infections have been reported; however, cross-species transmission, co-infection with multiple PKoV strains, and viral recombination events have all been documented [85, 88].

Porcine sapelovirus (PSV) is a non-enveloped, positive-sense single-stranded RNA virus that belongs to the genus Sapelovirus in the family Picornaviridae. PSV is most closely related to members of the Enterovirus genus and was formerly known as porcine enterovirus 8 (PEV-8), classified as porcine enterovirus A (PEV-A) [89]. PSV commonly results in asymptomatic infection of the gastrointestinal tract [90–93]. Pathogenic infections can lead to a variety of clinical syndromes including diarrhea, respiratory disease, reproductive disorders and polioencephalomyelitis [94–96].

Based on the blast tool for genetic similarity assessment and the phylogenetic analysis, we see that the Sapovirus sequences detected in our study show great similarity with Sapovirus sequences from Brazil and China (Fig. 5). The Kobuvirus sequences detected in our study, show close genetic relationship with Porcine Kobuvirus sequences from Belgium, Japan, Italy and USA (Fig. 6), while the Sapelovirus sequence that we obtained is closely genetically related with Sapelovirus A sequences from China and Germany (Fig. 7). As in the case of PAstV, these observations indicate the absence of barriers to gene flow of those virus strains, supporting a genetic admixture pattern for which similar explanations may also apply for the detection of strains that are closely genetically related with Sapovirus, Kobuvirus and Sapelovirus strains from those countries. Kobuvirus and Sapelovirus belong to the family Picornaviridae. This is not the first study where Kobuvirus, Sapelovirus and Astrovirus have been detected with p289-p290 primer pair. In Gábor Reuter et al. [97], Kobuvirus was detected in porcine stool samples examined for Caliciviruses with primers p289–p290. This study revealed that the conserved 3D motif of the YGDD amino acid (for which reverse primer p289 was designed) is also present in Kobuviruses. Based on the electrophoresis results, Sapovirus and Norovirus can be differentiated from Kobuvirus by the size of the amplicon. Sapovirus amplicons with p289–p290 primer pair have a size of 331 bp, Norovirus amplicons have a size of 319 bp, while Kobuvirus amplicons are sized 1065 bp. In Tibor Farkas et al. research in 2012 [82], Picornaviruses were accidentally detected in chicken and turkeys with the same primer pair. In Gábor Reuter et al. [98], detection of porcine Astrovirus and Kobuvirus with p289-p290 was also described. The RT-PCR product corresponding to Astrovirus was 720 bp sized. Comparison of p289 with the Astrovirus detected showed that the conserved region *RdRp* of the amino acid of YGDD motif of Caliciviruses (which primer p289 targets) is also common in Astroviruses. The results of all the aforementioned studies, are in line with the results in our study regarding the detection of Kobuvirus, Sapelovirus and Astrovirus with the primer pair p289-p290. The need for sequencing of the conventional RT-PCR amplicons is also confirmed by our results.

As far as the age groups in which Caliciviruses were detected are concerned, we see that finishing pigs was the main age group affected (based on both conventional and SYBR Green RT-PCR results).

## Conclusions

Molecular diagnosis of Astrovirus, Norovirus and Sapovirus is very challenging due to the great genetic variability of these RNA viruses. Due to this fact, the use of universal primers that target conserved regions of the viruses is necessary. Primer pairs PAstVDF-R and p289-p290 met these criteria. Comparison of conventional and SYBR Green RT-PCR methods showed that SYBR Green RT-PCR excels in terms of sensitivity. Therefore, SYBR Green RT-PCR can be a reliable tool for the first screening of samples in routine molecular detection of these RNA viruses, giving rapid, sensitive and specific results, especially in samples with extremely few viral load. Our study demonstrated the wide presence of Astrovirus in swine of all ages in Greece, as well as the presence of Sapovirus, mainly in finishing pigs, and Kobuvirus and Sapelovirus in suckling piglets and grower pigs respectively. Due to the fact that these viruses were detected to great extend in asymptomatic pigs, further research needs to be conducted in order to investigate the role of these viruses in disease from other viruses (in case of mixed infections) or their contribution to growth performance of swine. The wide presence of PAstV in swine population in Greece poses a necessity for investigating its pathogenicity as well as its surveillance potential. It should always be kept in mind that virus’s ability to mutate and the close contact of these animals with humans could trigger a zoonotic event in the future. From the present study it can be also concluded that all examined viruses occur in higher prevalence in fattening pigs. Further analysis is in progress to see the precise prevalence of the viruses in each age group. To our knowledge, this is the first molecular epidemiological study regarding Astrovirus, Norovirus and Sapovirus in pigs in Greece.

## Methods

### Sample collection

The population of pigs in Greece is estimated to approximately 1.3 million, with about 95% of them being bred intensively in 300 farrow-to-finish (FTF) pig farms with a size of more than 100 sows (Bouras personal communication).

For the purpose of the study, 24 pig herds were randomly selected from the entire Greek territory (8.0% of the Greek farms) based on their size (half smaller and half larger than 300 sows) in order to obtain representative data from the population herds. In addition, 4 establishments with less than 100 sows had been sampled.

During 2019, a total of 1400 stool samples were collected from different farms around Greece, including Region of Thrace, Macedonia, Epirus, Thessaly, Sterea Hellas, Peloponesse, and Crete (Fig. 1). From each farm, we collected 50 samples of faeces from pigs from five different age groups: suckling, post-weaning (nursery), growers, finishing pigs and sows. Ten samples (deriving from 10 different pigs housed in different pens) were essentially collected from each age group. The samples were collected with the use of swabs, which after the sampling were placed in disposable, sterile 1.5 ml Eppendorf (EP) tubes and transferred to the lab in isothermic boxes imbedded in ice. Each sample was re-suspended in 1 ml of phosphate-buffered saline (PBS), then vortexed for 5 min. After centrifugation at 12.000 g for 10 min, 40 μl of each supernatant was collected in pools of 5 (200 μl total volume), which were placed in new sterile 1.5 ml eppendorf tubes and stored at −80 °C until further processing. In this way, 280 pools were created from the original 1400 individual samples. Each pool contained samples from the same age group (2 pools from each age group per farm or 10 pools per farm). Thus, 56 pools were created in total for each age group.

### RNA extraction

Viral RNA was extracted from a volume of 200 μl per pooled sample, following the instructions of the RNA extraction kit “Cador pathogen kit” (Qiagen, Germany). After the RNA purification, the quantity and purity of the acquired RNA was measured using a Spectrophotometer (Eppendorf). The extracted RNA was stored at −80 °C until use (approximately two weeks).

### Reverse transcription PCR (RT-PCR)

#### Astrovirus

For the detection of porcine Astrovirus using conventional RT-PCR, the primers PAstVDF (5’-GAAKCRCTSYATGGGAARCTCCT-3’) and PAstVDR (5’-CTTTGGTCCKCCCCYCCAAA-3’) were used [60]. These primers target a conserved region within *ORF1b* of Astrovirus genome and produce an amplicon of 183 base pairs (bp). They are capable of detecting all five PAstV types. The QIAGEN OneStep RT-PCR Kit was used for the conventional PCR. Briefly, 4 μl of template RNA from each pooled sample were added to a mix of 10 μl 5 × QIAGEN OneStep RT-PCR Buffer, 2 μl dNTP Mix (final concentration 400 μM of each dNTP), 3 μl of each primer (final concentration 0.6 μM), 2 μl of QIAGEN OneStep RT-PCR Enzyme Mix, and 26 μl of RNase free water. Therefore, the total volume for the reaction was 50 μl. The conditions of the RT-PCR were the following: Reverse transcription was carried out at 50 °C for 30 min. Then the initial PCR activation step took place at 95 °C for 15 min followed by 40 cycles of denaturation for 30 s at 94 °C, annealing at 60 °C for 30 s and extension at 72 °C for 1 min. A final extension then took place at 72 °C for 10 min.

#### Norovirus and Sapovirus

Detection of Norovirus and Sapovirus in porcine fecal samples was performed in an RT-PCR using the same kit (QIAGEN OneStep RT-PCR Kit) and the universal calicivirus primer pair p289-p290 (p290: 5’-GATTACTCCAAGTGGGACTCCAC-3’—p289: 5’-TGACAATGTAATCATCACCATA-3’, [83]), targeting a conserved region of the *RdRp* of Caliciviruses, that creates an amplicon of 331 bp for Sapovirus and an amplicon of 318 bp for Norovirus. In this manner, simultaneous detection of both viruses was achieved. Briefly, 4 μl of template RNA from each pooled RNA purified sample were added in a mix containing 10 μl 5 × QIAGEN OneStep RT-PCR Buffer, 2 μl dNTP Mix (final concentration 400 μM of each dNTP), 3 μl of each primer (final concentration 0.6 μM), 2 μl of QIAGEN OneStep RT-PCR Enzyme Mix, and 26 μl of RNase free water. The reaction was conducted under the following conditions: The reverse transcription at 50 °C for 30 min was followed by an initial PCR activation step at 95 °C for 15 min and then by 40 cycles of 30 s at 94 °C, 1 min at 50 °C and 1 min at 72 °C, and a final extension step at 72 °C for 10 min.

PCR products of both PCR assays were stained with ethidium bromide and visualized after electrophoresis on a 1.5% agarose gel using UV light.

### SYBR-Green Real time RT-PCR

#### Astrovirus

SYBR-Green real time RT-PCR for the detection of porcine Astrovirus was performed with the same primer pair (PAstVDF- PAstVDR) utilized in the conventional RT-PCR. The Fast Gene IC Green One Step Mix kit (Nippon Genetics) was used for the reactions. One μl of template RNA was added to a mix of 10 μl of 2X FastGene® IC Green One Step Mix, 1 μl 20X FastGene® Scriptase, 0.8 μl of each primer of concentration 10 μΜ, and 6.4 μl of RNase free water. The conditions of the PCR were the following: 10 min at 45 °C, 2 min at 95 °C, followed by 40 cycles of 5 s at 95 °C and 1 min at 60 °C. Melt curve analysis was performed after these steps at a resolution from 55 °C to 95 °C with signal acquirement measurement every 5 s.

#### Norovirus and Sapovirus

The primer pair p289–p290 was also utilized in the SYBR-Green real time RT-PCR for the detection of Norovirus and Sapovirus in pigs. Their use in this method was as described in Mauroy’s et al. [79]. For the conduction of the PCR the same PCR kit was used (Fast Gene IC Green One Step Mix, Nippon Genetics). One μl of template RNA was added to a mix of 10 μl of 2X FastGene® IC Green One Step Mix, 1 μl 20X FastGene® Scriptase, 0.8 μl of each primer (400 nM final concentration), and 6.4 μl of RNase free water. The conditions of the PCR were the following: 10 min at 45 °C, 2 min at 95 °C, followed by 40 cycles of 5 s at 95 °C and 30 s at 51 °C. Melt curve analysis was performed after these steps as described above.

#### Sequencing

In an effort to validate the positive Astrovirus and Caliciviruses samples as indicated by the method of conventional RT-PCR, as well as scoping to phylogenetically analyze these samples, a randomly selected subsample was sent for purification and sequencing to Eurofins Scientific (Luxembourg). Sanger sequencing was performed bidirectionally for each PCR product in two runs, using forward and reverse primers of each PCR. Obtained sequences were read, edited and aligned in MEGA-X software package [99]. Nucleotide similarity with NCBI genetic database was assessed using the MEGABLAST search tool for highly similar sequences, embedded in the NCBI website (available at http://www.ncbi.nlm.nih.gov/blast/Blast.cgi). Phylogenetic relationships of the newly described haplotypes in comparison with corresponding ones, closely genetically related as assessed by the BLAST tool, obtained from the GenBank database, were evaluated in MEGA-X software. Particularly, sequences from GenBank, with over 90% similarity with the newly described ones in the present study were included in the analysis. Phylogenetic trees were constructed by both neighbor-joining analysis and Maximum Likelihood method. The confidence values of the internal nodes were calculated by performing bootstrap analyses with 1000 iterations. Particularly for Astrovirus characterized sequences, evolutionary genealogy was additionally estimated and depicted in a median-joining haplotype network, constructed in the software PopART [100].

## Supplementary Information

**Additional file 1**. Name, detected virus(es), sampling site, animal host age and GenBank accession number for all analyzed samples. All samples derived from asymptomatic animals.

## Data Availability

All data generated or analyzed during this study are included in this published article and its supplementary information files.

## Abbreviations

PAstV: Porcine Astrovirus
PoNoV: Porcine Norovirus
PoSaV: Porcine Sapovirus
RT-PCR: Reverse Transcription Polymerase Chain Reaction
ORF: Open: Reading frame
RdRp: RNA-dependent RNA polymerase
