# Supplementary material for: Epidemiology of Astrovirus, Norovirus and Sapovirus in Greek pig farms indicates high prevalence of Mamastrovirus suggesting the potential need for systematic surveillance
Source: Porcine Health Manag. 2022 Jan 9;8:5. doi: 10.1186/s40813-021-00245-8 (PMC8744241; doi:10.1186/s40813-021-00245-8)
Supplement: Supplementary file 1 — Additional file 1. Name, detected virus(es), sampling site, animal host age and GenBank accession number for all analyzed samples. All samples derived from asymptomatic animals. [file 40813_2021_245_MOESM1_ESM.docx]

**Additional file 1.** Name, detected virus(es), sampling site, animal host age and GenBank accession number for all analyzed samples. All samples derived from asymptomatic animals.

| **SAMPLE STATUS** | **SAMPLE NAME** | **CLINICAL STATUS** | **MELTING TEMPERATURE** | **SYBR Green**  **real time**  **RT-PCR** | **Conventional**  **RT-PCR** | **Sequencing** | **Age** | **Area where the positive sample was detected** | **GenBank accession numbers** |
| --- | --- | --- | --- | --- | --- | --- | --- | --- | --- |
| SaV + | X23AP10 | ASYMPTOMATIC | 85°C | + | + | Porcine sapovirus | post-weaning | Larisa | OK086800 |
| SaV+,  PAstV+ | X13PRO2 | ASYMPTOMATIC | 85°C | + | + | Porcine sapovirus,  Porcine Astrovirus | pre-fattening | Crete | OK066011(PAstV),  OK086801(PoSaV) |
| SaV+,  PAstV+ | X12AP1 | ASYMPTOMATIC | 85°C | + | + | Porcine sapovirus, Porcine Astrovirus | post-weaning | Larisa | OK066014(PAstV), |
| SapelovirusA+,  PAstV+ | X13201.2 | ASYMPTOMATIC | 85°C | + | + | Sapelovirus A,  Porcine Astrovirus | fattening | Crete | OK086799(Sapelovirus) |
| PoKoV+ | X14THIL1 | ASYMPTOMATIC | 85°C | + | + | Porcine Kobuvirus | Suckling | Sparta | OK086794 |
| PoKoV+ | X14THIL2 | ASYMPTOMATIC | 85°C | + | + | Porcine Kobuvirus | Suckling | Sparta | OK086796 |
| PoKoV+,  PAstV+ | X3THIL2 | ASYMPTOMATIC | 85°C | + | + | Porcine Kobuvirus,  Porcine Astrovirus | Suckling | Metsovo | OK066033(PAstV),  OK086798(PoKoV) |
| PAstV+ | X7AP1 | ASYMPTOMATIC | 85°C | + | + | Porcine Astrovirus | post-weaning | Chalkis | OK066007 |
| PAstV+ | X7PAX1 | ASYMPTOMATIC | 85°C | + | + | Porcine Astrovirus | fattening | Chalkis | OK066008 |
| PAstV+ | X7PRO1 | ASYMPTOMATIC | 85°C | + | + | Porcine Astrovirus | pre-fattening | Chalkis | OK066009 |
| PAstV+ | X7XOIR1 | ASYMPTOMATIC | 85°C | + | + | Porcine Astrovirus | sows | Chalkis | OK066010 |
| PAstV+ | X13AP2 | ASYMPTOMATIC | 85°C | + | + | Porcine Astrovirus | post-weaning | Crete | OK066012 |
| PAstV+ | X5XOIR | ASYMPTOMATIC | 85°C | + | + | Porcine Astrovirus | sows | Preveza | OK066013 |
| PAstV+ | X26AP2 | ASYMPTOMATIC | 85°C | + | + | Porcine Astrovirus | post-weaning | Larisa | OK066015 |
| PAstV+ | X26PAX1 | ASYMPTOMATIC | 85°C | + | + | Porcine Astrovirus | fattening | Larisa | OK066016 |
| PAstV+ | X23AP2 | ASYMPTOMATIC | 85°C | + | + | Porcine Astrovirus | post-weaning | Larisa | OK066017 |
| PAstV+ | X23PRO1 | ASYMPTOMATIC | 85°C | + | + | Porcine Astrovirus | pre-fattening | Larisa | OK066018 |
| PAstV+ | X23THIL1 | ASYMPTOMATIC | 85°C | + | + | Porcine Astrovirus | Suckling | Larisa | OK066019 |
| PAstV+ | X23XOIR1 | ASYMPTOMATIC | 85°C | + | + | Porcine Astrovirus | sows | Larisa | OK066020 |
| PAstV+ | X279 | ASYMPTOMATIC | 85°C | + | + | Porcine Astrovirus | fattening | Pieria | OK066021 |
| PAstV+ | X22XOIR | ASYMPTOMATIC | 85°C | + | + | Porcine Astrovirus | sows | Arkadia | OK066022 |
| PAstV+ | X11THIL1 | ASYMPTOMATIC | 85°C | + | + | Porcine Astrovirus | Suckling | Chalkidiki | OK066023 |
| PAstV+ | X11XOIR1 | ASYMPTOMATIC | 85°C | + | + | Porcine Astrovirus | sows | Chalkidiki | OK066024 |
| PAstV+ | X4PAX | ASYMPTOMATIC | 85°C | + | + | Porcine Astrovirus | fattening | Pieria | OK066025 |
| PAstV+ | X17XOIR | ASYMPTOMATIC | 85°C | + | + | Porcine Astrovirus | sows | Kozani | OK066026 |
| PAstV+ | X1AP1 | ASYMPTOMATIC | 85°C | + | + | Porcine Astrovirus | post-weaning | Larisa | OK066027 |
| PAstV+ | X16PAX1 | ASYMPTOMATIC | 85°C | + | + | Porcine Astrovirus | fattening | Kozani | OK066028 |
| PAstV+ | X16AP1 | ASYMPTOMATIC | 85°C | + | + | Porcine Astrovirus | post-weaning | Kozani | OK066029 |
| PAstV+ | X16PRO1 | ASYMPTOMATIC | 85°C | + | + | Porcine Astrovirus | pre-fattening | Kozani | OK066030 |
| PAstV+ | X3AP1 | ASYMPTOMATIC | 85°C | + | + | Porcine Astrovirus | post-weaning | Metsovo | OK066031 |
| PAstV+ | X3PAX1 | ASYMPTOMATIC | 85°C | + | + | Porcine Astrovirus | fattening | Metsovo | OK066032 |
| PAstV+ | X211 | ASYMPTOMATIC | 85°C | + | + | Porcine Astrovirus | fattening | Kilkis | OK066034 |
| PAstV+ | X191 | ASYMPTOMATIC | 85°C | + | + | Porcine Astrovirus | fattening | Kilkis | OK066035 |
| PAstV+ | X6PAX1 | ASYMPTOMATIC | 85°C | + | + | Porcine Astrovirus | fattening | Agrinio | OK066036 |
| PAstV+ | X28PAX | ASYMPTOMATIC | 85°C | + | + | Porcine Astrovirus | fattening | Pieria | OK066037 |
| PAstV+ | X28THIL | ASYMPTOMATIC | 85°C | + | + | Porcine Astrovirus | suckling | Pieria | OK066038 |
| PAstV+ | X28XOIR | ASYMPTOMATIC | 85°C | + | + | Porcine Astrovirus | sows | Pieria | OK066039 |
| PAstV+ | X14AP1 | ASYMPTOMATIC | 85°C | + | + | Porcine Astrovirus | Post-weaning | Sparta | OK066040 |
| PAstV+ | X14PAX1 | ASYMPTOMATIC | 85°C | + | + | Porcine Astrovirus | fattening | Sparta | OK066041 |
| PAstV+ | X15AP1 | ASYMPTOMATIC | 85°C | + | + | Porcine Astrovirus | Post-weaning | Ileia | OK066042 |
| PAstV+ | X15THIL1 | ASYMPTOMATIC | 85°C | + | + | Porcine Astrovirus | suckling | Ileia | OK066043 |
| PAstV+ | X9AP | ASYMPTOMATIC | 85°C | + | + | Porcine Astrovirus | Post-weaning | Karditsa | OK066044 |
| PAstV+ | X9PAX | ASYMPTOMATIC | 85°C | + | + | Porcine Astrovirus | fattening | Karditsa | OK066045 |
| PAstV+ | X9PRO | ASYMPTOMATIC | 85°C | + | + | Porcine Astrovirus | Pre-fattening | Karditsa | OK066046 |
| PAstV+ | X10XOIR1 | ASYMPTOMATIC | 85°C | + | + | Porcine Astrovirus | sows | Chalkis | OK066047 |
| PAstV+ | X10PAX1 | ASYMPTOMATIC | 85°C | + | + | Porcine Astrovirus | fattening | Chalkis | OK066048 |
